# Machine learning-based high-specificity diagnostic model for *Talaromyces marneffei* infection in febrile patients using routine clinical laboratory data

**DOI:** 10.3389/fmicb.2025.1654918

**Published:** 2025-09-04

**Authors:** Yingjun Xiao, Xiling Chen, Xiping Ou, Zheqing Dong, Xiaoyan Zhang, Wei Liang, Xiaojing Nan, Chan Xu, Xiaobo Lai, Peng Xu, Kui Fang

**Affiliations:** ^1^The Third Affiliated Hospital of Zhejiang Chinese Medical University, Hangzhou, Zhejiang, China; ^2^The Third School of Clinical Medicine, Zhejiang Chinese Medical University, Hangzhou, Zhejiang, China; ^3^The First People’s Hospital of Yuhang District, Hangzhou, Zhejiang, China; ^4^Luqiao Hospital of Traditional Chinese Medicine, Taizhou, Zhejiang, China; ^5^School of Medical Technology and Information Engineering, Zhejiang Chinese Medical University, Hangzhou, Zhejiang, China

**Keywords:** *Talaromyces marneffei*, febrile patients, machine learning, predictive model, feature mining

## Abstract

**Objective:**

This study developed and validated a machine learning (ML)-based predictive model utilizing febrile patients’ routine clinical laboratory data for the purpose of screening such patients for *Talaromyces marneffei* infection and to provide reference information for feature selection in the subsequent establishment of a more precise early warning model.

**Methods:**

This retrospective study enrolled febrile patients who visited Zhejiang Provincial People’s Hospital and the Third Affiliated Hospital of Zhejiang Chinese Medical University from January 2021–April 2025. Patient data, including sex, age, and laboratory test results, were collected. Through sparse partial least squares discriminant analysis, the most informative features were extracted from the dataset. Six classic machine learning algorithms were utilized to develop the optimal predictive model through 1000 bootstrap resamplings. Finally, the model was validated on an independent clinical validation dataset.

**Results:**

The training dataset comprised 485 febrile patients (141 with *T. marneffei* infection). The clinical validation dataset comprised 1,953 febrile patients (13 with *T. marneffei* infection). The random forest model demonstrated the highest performance in classifying *T. marneffei*-infected patients, with an area under the receiver operating characteristic curve of 0.987 in out-of-bag validation and 0.989 in clinical validation. The model also exhibited good specificity (0.999) for *T. marneffei* infection and good sensitivity (0.845) in predicting bacteraemia in clinical validation.

**Conclusion:**

A random forest model can effectively utilize routine clinical laboratory data to predict *T. marneffei* infection and bacteraemia in febrile patients, offering a promising early screening tool for individuals at high risk for *T. marneffei* infection.

## Introduction

*Talaromyces marneffei* (*T. marneffei*), formerly known as *Penicillium marneffei*, is a thermally dimorphic fungus belonging to the genus Talaromyces. Upon phagocytosis by macrophages within a mammalian host (at 37 °C), the spores of this fungus exhibit resistance to oxidative stress and nutritional deprivation, undergoing a transformation into fission yeast (Boyce et al., 2018). This characteristic renders it an opportunistic pathogen that primarily infects immunocompromised individuals. Previous reports have focused predominantly on HIV-infected populations. However, the proportion of non-HIV-coinfected patients with *T. marneffei* infection is increasing annually worldwide (Chan et al., 2016; Li et al., 2021). These non-HIV-coinfected patients include those who are receiving immunosuppressive therapy or have immunodeficiency disorders, and their mortality rate ranges from 24% to 51% because of misdiagnosis and agnostic delays (Li et al., 2024; Ly et al., 2020). Increasing the detection rate during the initial consultation while shortening the diagnostic time is crucial for reducing the rate of fatalities caused by *T. marneffei*.

Several key factors contribute to the high misdiagnosis rate and agnostic delays. First, fever is observed in almost all patients, and approximately half of them present with cough, while some may develop skeletal/joint lesions and skin/subcutaneous lesions (Li et al., 2023; Qiu et al., 2015). However, associated symptoms (e.g., umbilicated skin lesions) are relatively uncommon, making it easy to confuse *T. marneffei* infection with diseases such as tuberculosis and respiratory pathogen infections (Chan et al., 2016). Second, owing to the lack of vigilance among clinicians toward *T. marneffei* in nonendemic regions, this fungus is often first detected through blood cultures (You et al., 2021). However, blood cultures for *T. marneffei* detection take 7–14 days and have only 76% sensitivity, leading to missed diagnoses and delayed treatment (Ning et al., 2018). In contrast, bone marrow cultures and molecular or immunological detection techniques targeting the MP1 antigen can increase sensitivity to 90%–100% (Chen et al., 2022; Ling et al., 2022), but these tests require relevant clinical evidence for support.

Machine learning has been demonstrated to significantly increase accuracy in the clinical diagnosis of pathogen infections (Radaelli et al., 2024). Huang et al.’s (2022) employed a regression model in an HIV-infected population and identified key predictors useful for the differential diagnosis of *T. marneffei* infection, such as leukocytes and lactate dehydrogenase; together, these factors achieved an AUC of 0.815. Using a logistic regression model, Qiu et al. (2025) identified multiple independent predictors of *T. marneffei* infection in non-HIV-infected patients; these factors, including, among others, age and white blood cell differential, also jointly achieved an AUC of 0.9. These two pivotal studies indicate that *T. marneffei* infection can be predicted via routine blood cell counts, biochemical tests, and other conventional laboratory data. However, these studies were conducted in regions where *T. marneffei* is endemic (Guangdong and Guangxi, China), and the patient populations were stratified on the basis of HIV infection status. In nonendemic regions, patients often present with persistent fever as the primary symptom, and clinicians generally do not inquire about sensitive questions such as HIV infection status. For such complex patient populations, rapid alerts for *T. marneffei* infection on the basis of routinely available test results would hold especially high clinical value.

The objective of this study was to develop a predictive model for *T. marneffei* infection using routine laboratory test data from infected patients (including HIV-coinfected, non-HIV-coinfected patients, and patients whose HIV infection status is unclear), thereby significantly advancing the timing of clinical intervention and reducing the risk of patient mortality. Additionally, this study aimed to identify high-value predictive factors for future large-scale, multiregional, multicentre clinical trials of early warning models for *T. marneffei* infection.

## Materials and methods

### Patients and data collection

The patient data utilized in this study were retrospectively collected from febrile patients who visited the Third Affiliated Hospital of Zhejiang Chinese Medical University (Zhejiang, China), The First People’s Hospital of Yuhang District (Zhejiang, China), and Luqiao Hospital of Traditional Chinese Medicine (Zhejiang, China) between April 2021 and April 2025. The inclusion criteria for patients were as follows: (1) They met the diagnostic criteria for fever (as defined in the IDSA/SCCM consensus guidelines) (O’Grady et al., 2023). (2) They had undergone blood culture tests (because none of the hospitals involved in this research had a unified standard for blood culture, only patients who met the testing conditions outlined in the American Society for Microbiology Cumitech were selected). (3) Clinical information and laboratory test results, including blood culture, routine blood tests, biochemical tests, and procalcitonin measurement, were available. Anonymized patient information and test data were collected through the Laboratory Information System (LIS). The basic patient information included sex and age. The laboratory test data included blood culture results, routine blood tests, biochemical tests, and procalcitonin. To ensure the comparability of data from different institutions, all clinical research centers selected for this study employed a combination of mass spectrometry and biochemical methods to identify the pathogens in all positive blood cultures. The mass spectrometers used by all centers were all Autobio MS series fully automated microbial mass spectrometry detection systems and utilized the same pathogen identification database, which includes the spectral patterns of *T. marneffei*. The patients were divided into three groups, namely, *T. marneffei* infection, other pathogen infection (positive), and no pathogens detected (negative), according to the results of blood cultures. Data processing and modeling in this study were conducted within the R computing environment (version 4.4.2).

### Data processing

Samples and laboratory tests with more than 10% of values missing in any group were excluded to mitigate the impact of missing data on subsequent analyses. For the remaining samples with missing values, the advanced multiple imputation by chained equations (MICE) method was employed for imputation. The number of imputations was set at 5 to enhance the stability and accuracy of the imputation results. To eliminate the potential effects of different scales among various indicators and the influence of extreme values on model construction, all continuous variables underwent logarithmic transformation and Z-score standardization, ensuring the comparability of indicators within the model.

### Feature selection

All samples that tested positive for *T. marneffei* from January 2021 to September 2024, alongside randomly selected samples with positive and negative blood culture results, were used as the training dataset. Feature extraction was accomplished by tuning and establishing a sparse partial least squares discriminant analysis (sPLS-DA) via the mixOmics package (version 6.26.0). All analyses were conducted via R software (version 4.4.2). During the tuning process, the optimal number of components and the optimal number of variables (clinical and laboratory data) within each component were determined through a grid search that explored all possible parameter combinations. The top 2 inflection points (points with a second derivative of zero) were calculated on the basis of the trend and magnitude of changes in contribution and stability. The purpose of this step was to stratify features according to their contribution or stability and assist in further optimizing the number of features.

### Modeling and OOB validation

The performance of the features was validated via six classic algorithms, namely, the decision tree, random forest, neural network, conditional inference tree, C5.0 decision tree, and support vector machine algorithms (using the caret package, version 6.0.94). The validation process comprised 1,000 bootstrap resampling iterations. For each sample in the original dataset, we collected its predictions whenever it appeared in out-of-bag (OOB) validation sets. The final confusion matrix was generated by aggregating predictions and comparing with the true class labels across all samples. From the confusion matrices, various performance metrics, such as the accuracy, precision, recall, and F1 score, were calculated to assess the model’s classification effectiveness comprehensively. Receiver operating characteristic (ROC) curves and area under the curve (AUC) values were used to evaluate the performance of the validation models. To analyze the model’s classification performance for each category via ROC curves, we transformed the ternary (three-class) classification problem into three binary (two-class) classification problems (*T. marneffei* vs. non-*T. marneffei*, positive vs. nonpositive, etc.).

### Validation of the optimal model in a clinical environment

To detect potential sampling errors during the generation of the training dataset and validate the model’s performance in real-world clinical practice, we applied the optimal prediction model continuously to all eligible samples collected between October 2024 and March 2025. This approach was employed to assess the model’s classification capability in authentic clinical scenarios. The model was evaluated according to the same indices mentioned above. Given that other species of fungi are prone to being confused with *T. marneffei* during clinical diagnosis, we specifically extracted the prediction results of fungal-infected cases to evaluate the model’s performance in correctly identifying them.

### Statistical methods

The Games–Howell method was employed for significance testing of features across different groups, accommodating data that were not normally distributed and exhibited unequal variances. The Holm–Bonferroni method was used to adjust the *P*-values for multiple comparisons. In terms of model performance comparison, the DeLong test was employed to conduct significance tests on the differences in AUCs among different groups of models, ensuring that the comparison results of model performance were statistically meaningful. Data distributions are presented as medians and 95% confidence intervals (95% CIs).

## Results

### Overview training data

**FIGURE 1:**
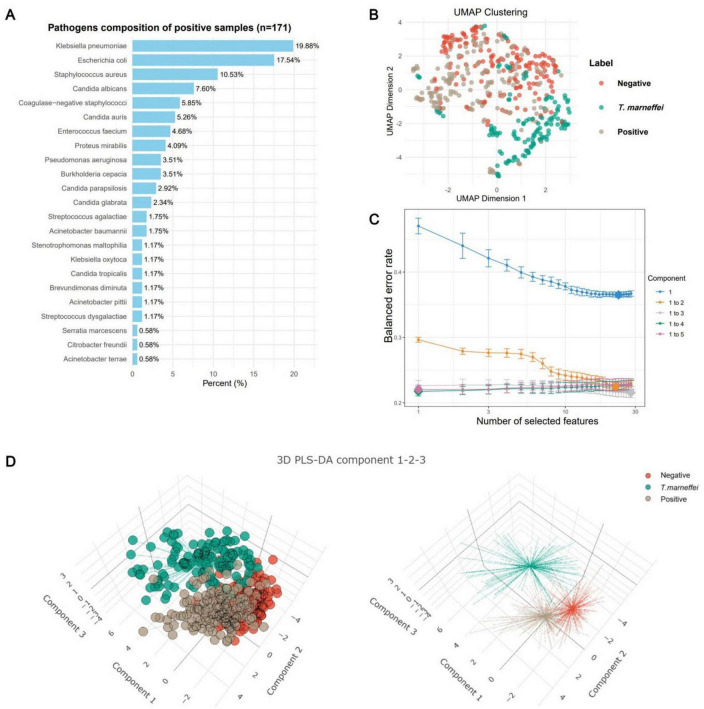
Characteristics of the training sample data. **(A)** Frequency plot of pathogen species in patients with positive blood cultures. **(B)** Uniform manifold approximation and projection (UMAP) clustering plot of patients (*n* = 485) in the training dataset. The clustering plot utilized all 28 eligible laboratory tests (including sex and age). Patients with *T. marneffei* infection (*n* = 141) presented distinct clustering boundaries, whereas the boundaries for blood-culture-negative (*n* = 173) and blood-culture-positive (*n* = 171) patients were less pronounced. **(C)** Error convergence curve of the sparse partial least squares discriminant analysis (sPLS-DA) model during the calculation of its optimal parameters. **(D)** Clustering analysis of samples based on the first three components of the sPLS-DA model, yielding results similar to those of the UMAP clustering.

From January 2021 to March 2025, 37,063 febrile patients (inpatients and outpatients) met the inclusion criteria, including 141 with *T. marneffei* infections, 4,968 with positive blood cultures, and 31,954 with negative cultures. A total of 28 test items were retained. To ensure balanced training data and meet machine learning requirements (number of samples > 10× number of features), all 141 *T. marneffei*-infected patients (2021–2024) and random subsets of patients with positive and negative blood culture (171 and 173, respectively) were included (the raw data are presented in Supplementary Table 1, and the summary is provided in Supplementary Table 2). The training cohort comprised 70.3% males (*n* = 341) and 29.7% females (*n* = 144) aged 19–102 years (median = 67 years; 95% CI: 27–94). Additional data on the pathogen species and intergroup differences are shown in Figure 1A and Supplementary Figure 1, respectively. UMAP clustering revealed distinct groupings, with clear separation between *T. marneffei* and the other groups (Figure 1B).

### Feature selection

The sPLS-DA algorithm identifies feature contributions by fitting an optimal classification model. With 30 iterations, the model error decreased until three components minimized it, beyond which overfitting occurred (Figure 1C). Thus, we used the first three components for analysis. Clustering revealed distinct groups, although the negative and positive clusters overlapped, indicating good *T. marneffei* detection but limited differentiation between blood-culture-negative and blood-culture-positive cases (Figure 1D).

### Feature analysis

**FIGURE 2:**
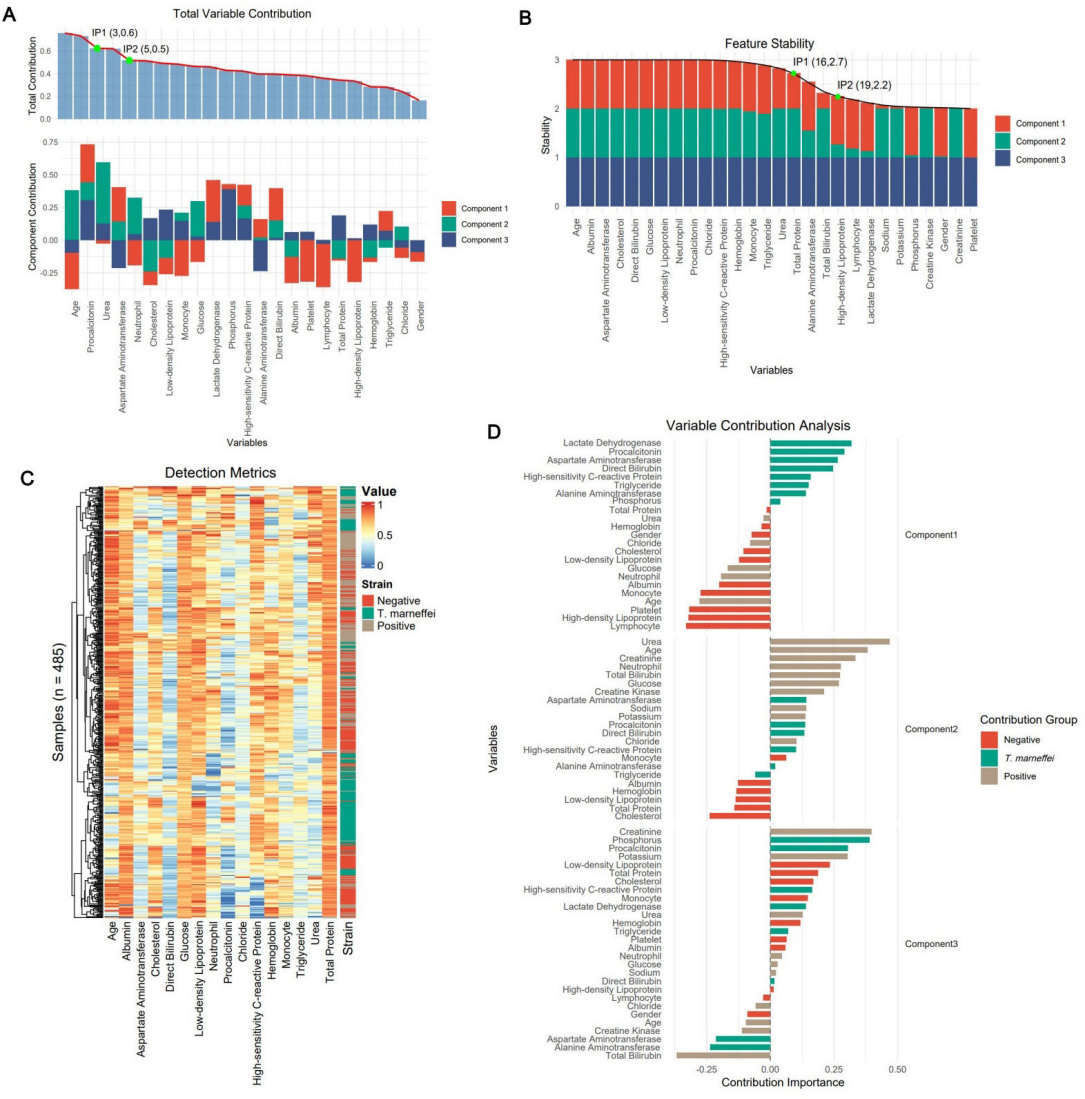
Extraction of the optimal feature set via sPLS-DA. **(A)** The subplots above and below represent the total contribution of features and the contribution of features within each component, respectively. The green dots represent the inflection points of the contribution trend line (the preceding numbers denote the feature indices, and the subsequent numbers represent the fitted values of the trend line). **(B)** Component-based total stability of features. **(C)** Sample-based clustering heatmap of the 16 optimal features selected on the basis of feature stability. The colors represent the results of various laboratory tests. **(D)** Contributions of group-based features.

**FIGURE 3:**
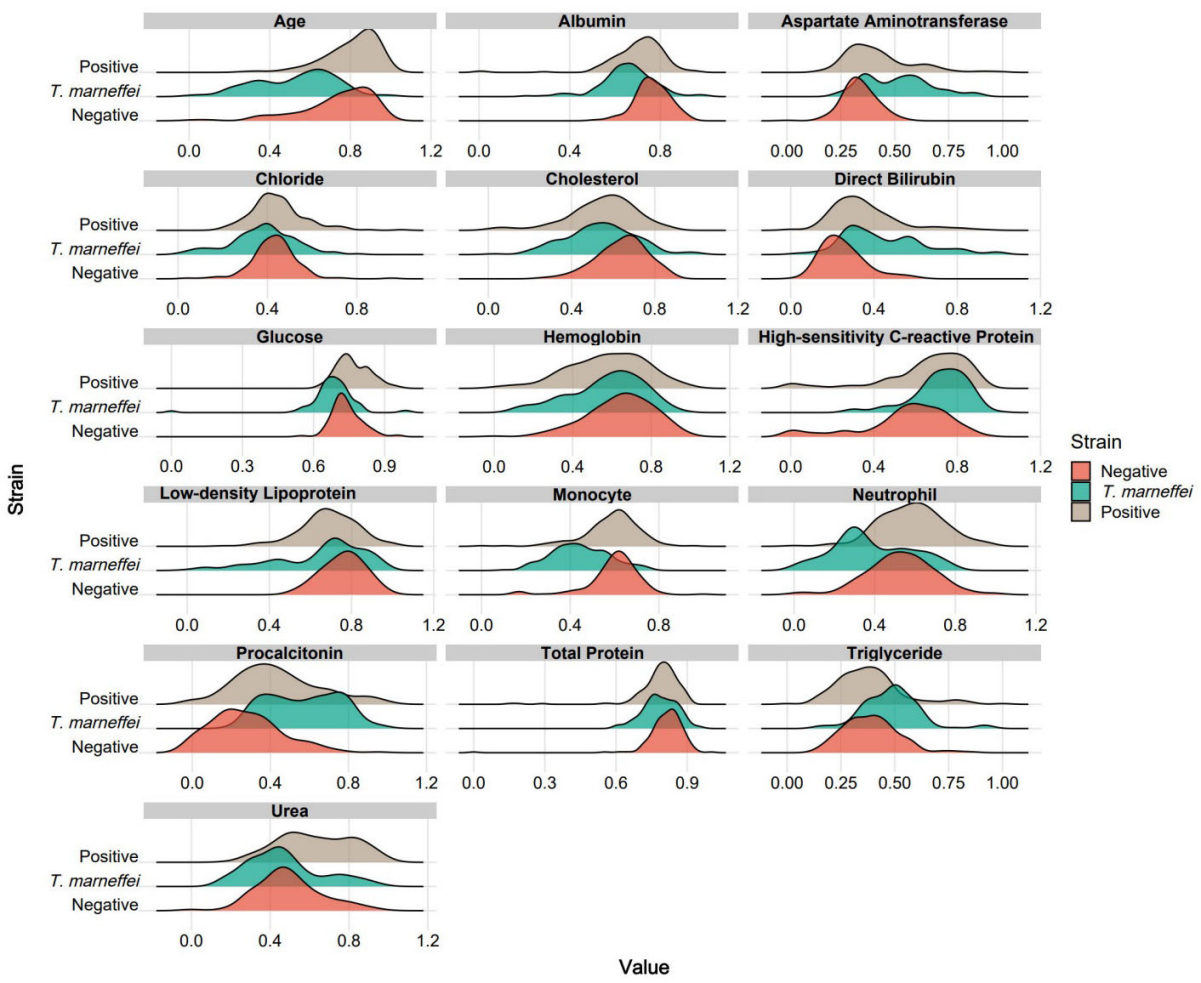
Ridge plots for each group based on the 16 optimal features. For clearer visual comparison, all the results in the plots were subjected to logarithmic transformation and normalization.

By analyzing feature contribution and stability in the sPLS-DA model, we plotted ranking diagrams for total contribution (Figure 2A) and stability (Figure 2B). Significant contribution changes occurred at the 3rd and 5th features, and robustness changes occurred at the 16th and 19th features. For subsequent modeling, we selected the top 16 features ranked by stability because higher stability implies that these features make more consistent contributions across different data subsets and are more likely to be genuine features. Figure 2C shows a cluster heatmap of these 16 features, with patients aggregated well in one-dimensional space, especially *T. marneffei*-infected patients; the test items showed intuitive characteristics such as younger age and higher procalcitonin levels in *T. marneffei*-infected patients. Figure 2D shows the feature–group relationships for each component. Component 1 mainly distinguishes the *T. marneffei*-infected group, Component 2 mainly contributes to differentiating the negative and positive groups, and Component 3 supplements the first two. Ridge plots (Figure 3) clearly show the distributions of each feature: the *T. marneffei* group were younger and had lower monocyte and neutrophil counts but higher triglyceride and procalcitonin levels, etc.; blood-culture-positive patients were older and had higher urea nitrogen levels. Multiple items in the *T. marneffei* group had a bimodal or non-normal distribution, indicating that the patients in this group exhibited heterogeneity.

### Model performance

**FIGURE 4:**
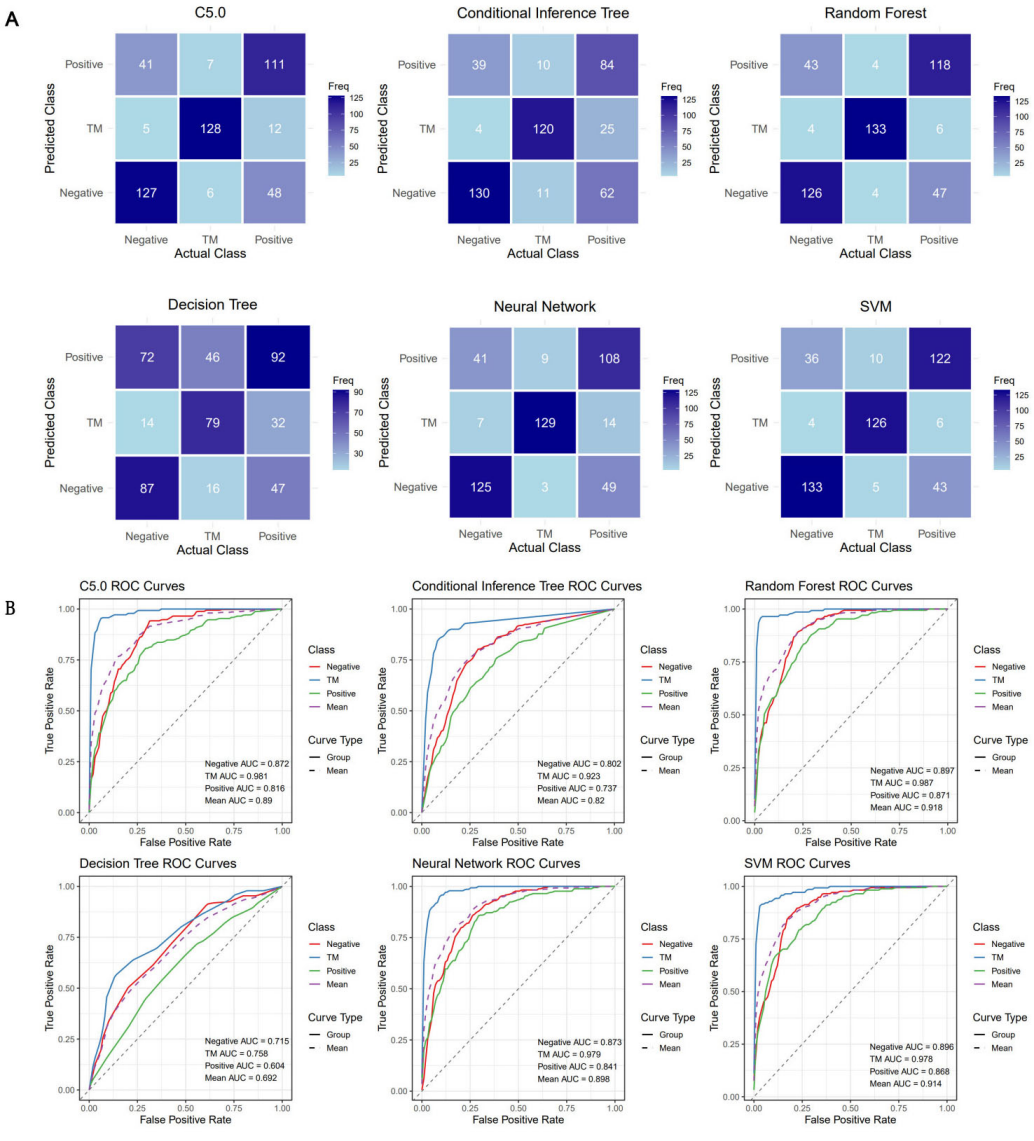
Six three-class classification models were constructed using the 16 optimal features. **(A)** Confusion matrices for each model were employed to calculate crucial evaluation indices, including the sensitivity, specificity, accuracy, and F1 score. **(B)** Receiver operating characteristic (ROC) curves for each model, along with the area under the curve (AUC) values for each class, were utilized to assess the classification performance of the models in a threshold-independent manner.

**FIGURE 5:**
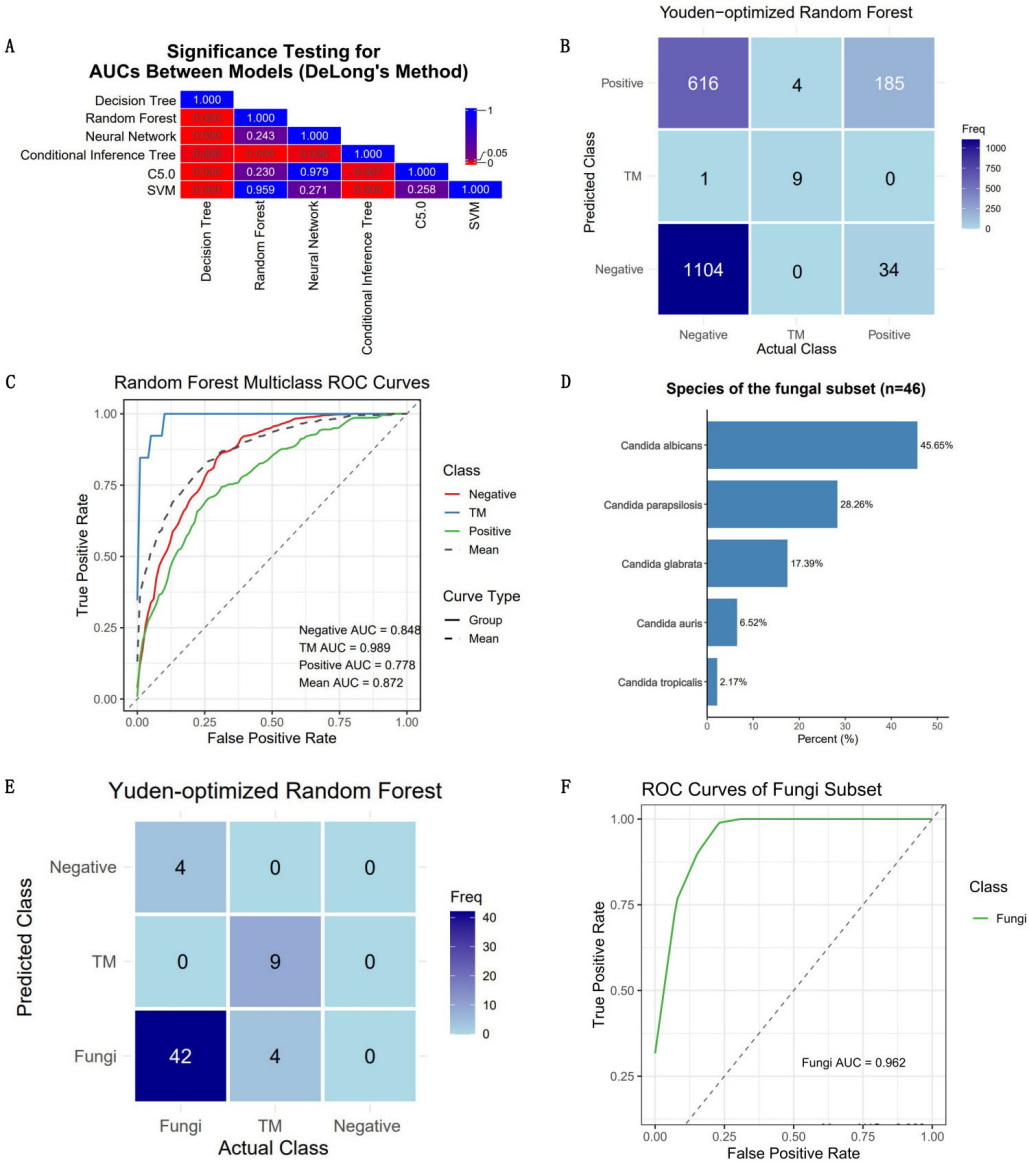
Clinical validation outcomes of the optimal model. The performance of the optimal model was rigorously evaluated using an independent and continuous dataset (*n* = 1,953). **(A)** The disparities in the mean AUC across the six models were utilized as the criterion for selecting the optimal model. The numbers in the grid represent *P*-values. **(B)** Confusion matrix for the clinical validation of the optimal model (random forest). **(C)** ROC curve of the optimal model, accompanied by AUC values for each category. The model achieved an average AUC of 0.872 for three-class classification, with the highest AUC (0.989) observed for predicting *T. marneffei*-infected patients. **(D)** The quantity and proportion of fungal patients in the validation dataset. **(E)** Confusion matrix of the model’s classification of fungi into the positive group in the validation dataset. **(F)** ROC curve of the model’s classification of fungi into the positive group in the validation dataset.

The confusion matrices of all the models are shown in Figure 4A. Table 1 was generated on the basis of the confusion matrices. Table 1 shows that the SVM model had the highest overall accuracy (0.786; 95% CI: 0.746–0.821), followed by random forest model (0.777; 95% CI: 0.738–0.814). In classifying the *T. marneffei* group, the random forest model had the highest accuracy (accuracy = 0.957), followed by the SVM model (accuracy = 0.932). Figure 4B shows that the random forest model had the highest average AUC (0.918), followed by the SVM model (0.914). Similarly, in classifying *T. marneffei*-infected patients, the random forest model had the highest AUC value (0.987), followed by the SVM model (0.978). There was also no significant difference in the average AUC between the two models (*P* = 0.959) (Figure 5A). Among all the models, the decision tree model had the worst performance in terms of both average AUC (0.692) and overall accuracy (0.532, 95% CI: 0.486–0.577). Since the main purpose of this study was to identify *T. marneffei*-infected patients, the random forest model was selected as the final model for subsequent clinical validation.

**TABLE 1:** The results of the six classical models established using optimized features.

Model	Random forest			Neural network			Conditional inference tree			SVM			Decision tree			C5.0 decision tree			Clinical validation			
Group	Neg	TM	Pos	Neg	TM	Pos	Neg	TM	Pos	Neg	TM	Pos	Neg	TM	Pos	Neg	TM	Pos	Neg	TM	Pos	Fungi
Sensitivity	0.728	0.943	0.69	0.723	0.915	0.632	0.751	0.851	0.491	0.769	0.894	0.714	0.503	0.56	0.538	0.734	0.908	0.649	0.642	0.692	0.845	0.913
Specificity	0.837	0.971	0.850	0.833	0.939	0.841	0.766	0.916	0.844	0.846	0.971	0.854	0.798	0.866	0.624	0.827	0.951	0.847	0.853	0.999	0.642	0.692
PPV	0.712	0.930	0.715	0.706	0.86	0.684	0.64	0.805	0.632	0.735	0.927	0.726	0.580	0.632	0.438	0.702	0.883	0.698	0.970	0.900	0.23	–
NPV	0.847	0.977	0.834	0.844	0.964	0.807	0.848	0.938	0.753	0.868	0.957	0.845	0.743	0.828	0.713	0.849	0.962	0.816	0.243	0.998	0.970	–
Precision	0.712	0.930	0.715	0.706	0.860	0.684	0.64	0.805	0.632	0.735	0.927	0.726	0.58	0.632	0.438	0.702	0.883	0.698	0.970	0.900	0.23	–
Recall	0.728	0.943	0.690	0.723	0.915	0.632	0.751	0.851	0.491	0.769	0.894	0.714	0.503	0.560	0.538	0.734	0.908	0.649	0.642	0.692	0.845	–
F1 score	0.720	0.937	0.702	0.714	0.887	0.657	0.692	0.828	0.553	0.751	0.910	0.720	0.539	0.594	0.483	0.718	0.895	0.673	0.772	0.783	0.361	–
Prevalence	0.357	0.291	0.353	0.357	0.291	0.353	0.357	0.291	0.353	0.357	0.291	0.353	0.357	0.291	0.353	0.357	0.291	0.353	0.881	0.007	0.112	–
DR	0.260	0.274	0.243	0.258	0.266	0.223	0.268	0.247	0.173	0.274	0.26	0.252	0.179	0.163	0.19	0.262	0.264	0.229	0.565	0.005	0.095	–
DP	0.365	0.295	0.340	0.365	0.309	0.326	0.419	0.307	0.274	0.373	0.28	0.346	0.309	0.258	0.433	0.373	0.299	0.328	0.583	0.005	0.412	–
Balanced accuracy	0.782	0.957	0.770	0.778	0.927	0.736	0.759	0.883	0.668	0.808	0.932	0.784	0.651	0.713	0.581	0.781	0.929	0.748	0.748	0.846	0.744	0.803
Accuracy (95% CI)	0.777 (0.738–0.814)			0.746 (0.705–0.785)			0.689 (0.645–0.730)			0.786 (0.746–0.821)			0.532 (0.486–0.577)			0.755 (0.713–0.792)			0.665 (0.643–0.686)			
Kappa	0.665			0.619			0.532			0.677			0.293			0.631			0.238			

SVM, support vector machine; DR, detection rate; DP, detection prevalence; PPV, positive predictive value; NPV, negative predictive value.

### Clinical validation

Data from all eligible febrile patients seen from January 2025 to March 2025 were collected. There were 1,953 fever patients in total, including 1,721 patients with negative blood cultures, 219 patients with positive blood cultures, and 13 patients infected with *T. marneffei* (a summary is provided in Supplementary Table 3). Since these data were normalized independently of the training dataset, the predicted probabilities were binarized using the Youden index as the threshold to obtain the predicted classification labels (Figure 5B). The results revealed that the overall accuracy of the model was 0.665 (95% CI: 0.643–0.686), and the kappa value was only 0.238, which might be due to the imbalance in the dataset (Table 1). The model had high specificity when separately predicting blood-culture-negative and *T. marneffei*-infected patients, with values of 0.853 and 0.999, respectively, but poor sensitivity, with values of 0.642 and 0.692, respectively. When predicting blood-culture-positive samples, the model had relatively good sensitivity (0.845) but poor specificity (0.642). The balanced accuracy (which corrects for the distortion of overall accuracy caused by dataset imbalance) of the model when separately distinguishing the positive, negative, and *T. marneffei* groups was 0.748, 0.846, and 0.744, respectively, indicating that the model had the best predictive ability for *T. marneffei*-infected patients. This trend was consistent with the AUC values of the ROC curves for the three categories, which were 0.848, 0.989, and 0.778, respectively (Figure 5C). The above results indicate that the model performed well in distinguishing cases of *T. marneffei* infection. Moreover, the AUC, sensitivity, specificity, and balanced accuracy of the model in classifying fungi and *T. marneffei* were 0.962, 0.913, 0.692, and 0.803, respectively (Table 1 and Figures 5D–F).

## Discussion

*T. marneffei* is predominantly distributed in Southeast Asia (including Vietnam, Thailand, and southern China), India, and southern China. Its conidia are transmitted primarily via aerosols (Wangsanut et al., 2023). Fever is the most prominent clinical manifestation of *T. marneffei* infection, with a prevalence rate exceeding 93% in both adults and children (Sun et al., 2021; Zeng et al., 2021). In clinical diagnosis, a history of exposure to endemic areas and HIV infection are crucial indicators suggesting *T. marneffei* infection (Patel et al., 2024). However, in nonendemic areas, clinicians may not initially consider *T. marneffei* infection when treating febrile patients and may not inquire about the aforementioned information. An important role of the clinical laboratory is to provide objective reference information for clinical decision-making. Therefore, in this study, we did not consider including subjective indicators such as patient history as training data for the model. The training data encompassed inpatients and outpatients from multiple clinical research centers, including both AIDS patients and non-AIDS patients, to ensure the diversity of the training dataset (the proportions of AIDS patients are detailed in Supplementary Tables 2, 3). A high diversity of training data can significantly improve a model’s generalization ability, strengthen its performance in real-world settings, and reduce bias in the extracted features that may arise from the use of a single sample source (Konkel et al., 2023; Zhang et al., 2023).

To date, studies on the differential diagnosis of *T. marneffei* infection using routine data (including clinical signs and laboratory tests) have been limited, with notable contributions from Lu et al. (2025), Qiu et al. (2025), and Huang et al.’s (2022). Lu et al. (2025) developed a combined model utilizing CT scans and clinical indicators, which serves as an effective assessment tool for distinguishing whether pulmonary infections in HIV patients are caused by *T. marneffei*. Huang et al.’s (2022) constructed a linear regression model by selecting multiple blood cell indicators, achieving an AUC of 0.815, with sensitivity and specificity of 0.762 and 0.761, respectively, in diagnosing HIV patients co-infected with *T. marneffei*. Additionally, Qiu et al. (2025) developed a logistic regression model incorporating blood cell indices, certain biochemical markers, and clinical symptoms, which attained an AUC of 0.918 (95% CI: 0.884–0.953) in differentiating pulmonary tuberculosis from *T. marneffei* infection in non-HIV patients.

Our model, which references the indicators from the above studies and incorporates additional inflammation-related and biochemical markers, demonstrated AUC values exceeding 0.98 in both OOB validation and clinical validation, outperforming the models by Qiu et al. (2025) and Huang et al.’s (2022) Specifically, our model achieved a specificity of 0.999 in the validation dataset, although its sensitivity was 0.692, which is lower than that of Huang et al.’s (2022) model. The imbalance between specificity and sensitivity may be attributed to the optimal Youden index selecting an imbalanced threshold to maximize the AUC. In practical application, this threshold can be adjusted according to clinical needs. Although our study did not separate HIV-infected patients from non-HIV-infected ones, which enhances the applicability of our model, our training dataset did not include tuberculosis patients. Consequently, the diagnostic efficacy of our model in differentiating tuberculosis from *T. marneffei* infection may not be on par with that of Qiu et al. (2025) model. Besides, during both OOB sample validation and clinical practice validation, our model demonstrated significant limitations in differentiating febrile patients with negative and positive blood cultures. There are likely two main reasons for this phenomenon. First, to ensure balance in the training dataset, we randomly selected a training dataset of 171 patients with positive blood cultures. Clearly, 171 positive samples cannot adequately cover the diversity of pathogen species. Similarly, the training data for negative patients failed to effectively encompass the characteristics of this group, which may be the primary reason for the model’s low accuracy in differentiating between negative and positive patients. Second, the insufficient number of representative features may be another important factor contributing to the model’s inability to efficiently distinguish between patients with negative and positive blood cultures (Sterkenburg, 2025).

Lu et al. (2025), Huang et al.’s (2022), and Qiu et al. (2025) identified key laboratory predictors such as aspartate transaminase (AST) and albumin levels; platelet and neutrophil counts. Despite differences in patient populations between our study and these previous studies, the feature analysis in our study also found that these indicators, including albumin, neutrophils, and AST, are the optimal features, underscoring their importance in predicting *T. marneffei* infection. Notably, the decrease in albumin and increase in AST in *T. marneffei*-infected patients (Supplementary Figure 1) have been confirmed in other relevant clinical studies (Li et al., 2016; Peng et al., 2022). Our findings also revealed an abnormal bidirectional (bimodal) distribution of neutrophils (Figure 3). Previous research has suggested that this phenomenon may be associated with HIV infection. In non-HIV-infected individuals, neutrophil counts tend to be elevated in the event of *T. marneffei* infection (Chen et al., 2021), whereas in HIV-infected patients, neutrophil counts decrease due to immunodeficiency (Li et al., 2016).

Furthermore, our machine learning model identified several new laboratory markers with potential predictive value for *T. marneffei* infection, including lactate dehydrogenase (LDH), procalcitonin (PCT), high-sensitivity C-reactive protein (hs-CRP), direct bilirubin (DB), and triglycerides (TG). We observed significantly elevated levels of these markers in *T. marneffei*-infected patients compared with blood-culture-negative febrile patients. Notably, LDH, DB, and TG were also significantly elevated in *T. marneffei*-infected patients compared with all other febrile patients. Except for TG, the elevation of these markers is supported by relevant clinical studies (Huang et al.’s, 2022; Li et al., 2024; Shi et al., 2021; Sun et al., 2021; Wang et al., 2024). An increased level of procalcitonin (PCT) is considered an independent risk factor for mortality in patients with *Talaromyces marneffei* infection (Sun et al., 2021). *T. marneffei* can induce hepatocyte pyroptosis, releasing large amounts of IL-1β and IL-18 (Ma et al., 2021; Wang et al., 2022), which may trigger hepatic inflammatory responses, potentially explaining the increases in LDH, DB, and TG.

Additionally, we identified laboratory indicators associated with predicting blood culture positivity (including bacteremia and fungemia), including urea, creatinine, age, total bilirubin, and neutrophil count. These markers were significantly elevated in our study compared with blood-culture-negative patients or compared with both blood-culture-negative and *T. marneffei*-infected patients. Clinical studies have shown that older patients are more prone to bacteraemia (da Silva et al., 2021). Elevated creatinine and urea levels suggest renal dysfunction, which may be associated with acute kidney injury commonly accompanying bacteraemia (Lentini et al., 2012). Moreover, elevated blood urea nitrogen is significantly correlated with bacteraemia prognosis (Salih et al., 2013). Elevated bilirubin levels may be related to specific bacterial infections or endotoxaemia (Azizoglu et al., 2024). During bacteraemia, neutrophils serve as the primary effector cells of innate immunity. Therefore, neutrophilia is a characteristic feature of bacteraemic patients (Azizoglu et al., 2024; Guo et al., 2023), and neutrophil counts are positively correlated with the bacterial load in the bloodstream (Han et al., 2023).

This study has several limitations. First, as previously mentioned, to ensure a balanced sample size across categories in the training dataset, the representativeness of the training samples for patients with negative and positive blood cultures was somewhat inadequate. Notably, fungi and mycobacteria were not explicitly trained or validated as independent output categories in our model. Consequently, when applied to the differential diagnosis of patients with suspected *T. marneffei* and fungi infection, the model may not demonstrate equivalent diagnostic accuracy in this specific patient population. Second, owing to the low number of *T. marneffei*-infected patients in nonendemic areas, the proportion of *T. marneffei*-infected patients in our clinical validation dataset was highly imbalanced. Consequently, metrics that are sensitive to data balance, such as the kappa value, may not hold high reference value in the clinical validation results. Finally, the study subjects were primarily from Zhejiang Province, China, and the distribution and clinical manifestations of *T. marneffei* infection in nonendemic areas may vary globally. Therefore, the applicability and generalizability of this model may be limited in other regions.

## Conclusion

Our study has successfully established a highly specific model for early screening and identification of blood-culture-positive and *T. marneffei*-infected febrile patients and also highlights a set of classification-related features. Furthermore, we validated the feasibility of efficiently providing an early warning of *T. marneffei* infection in febrile patients via routine laboratory data.

## Data Availability

The original contributions presented in this study are included in this article/Supplementary material, further inquiries can be directed to the corresponding authors.
