# Supplementary material for: Machine learning-based high-specificity diagnostic model for Talaromyces marneffei infection in febrile patients using routine clinical laboratory data
Source: Front Microbiol. 2025 Sep 4;16:1654918. doi: 10.3389/fmicb.2025.1654918 (PMC12443677; doi:10.3389/fmicb.2025.1654918)
Supplement: Supplementary file 1 [file Data_Sheet_1.pdf]

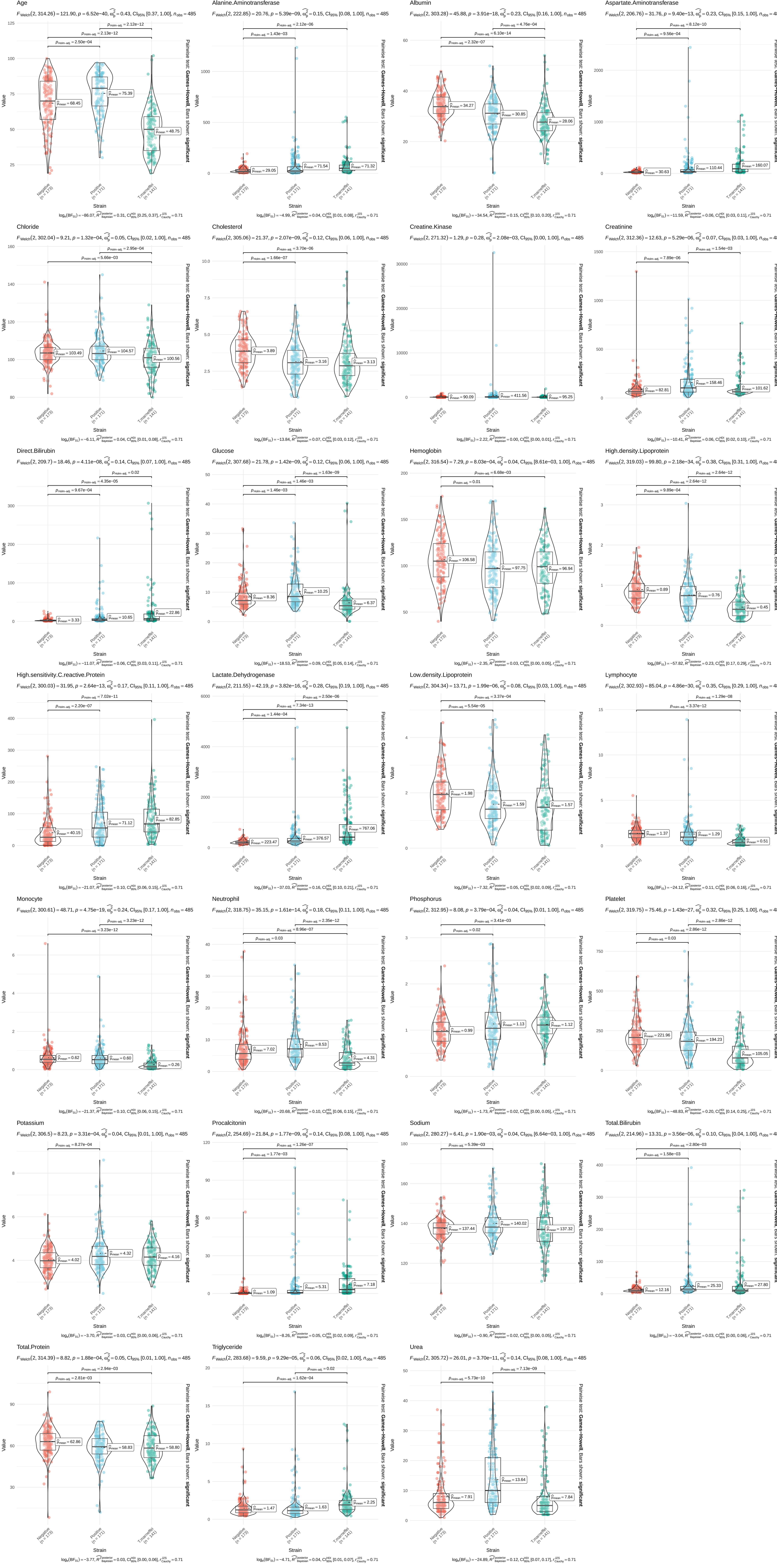

Figure 1: Comparisons of all features between groups. The upper and lower edges of each box represent the upper and lower quartiles, respectively, and the middle line represents the median. The violin plot is drawn on thebasis of thedata distribution. The mean is indicated by a yellow dot (this dot does not represent the metabolite abundance value). The adjusted p values for comparisons between groups are shown above when the adjusted p value is less than 0.05. The information from the Bayes factor analysis is located at the bottom.
